# Long-term Risk of Overdose or Mental Health Crisis After Opioid Dose Tapering

**DOI:** 10.1001/jamanetworkopen.2022.16726

**Published:** 2022-06-13

**Authors:** Joshua J. Fenton, Elizabeth Magnan, Irakis Erik Tseregounis, Guibo Xing, Alicia L. Agnoli, Daniel J. Tancredi

**Affiliations:** 1 Department of Family and Community Medicine, University of California, Davis, Sacramento; 2 Center for Healthcare Policy and Research, University of California, Davis, Sacramento; 3 Department of Internal Medicine, University of California, Davis, Sacramento; 4 Department of Pediatrics, University of California, Davis, Sacramento

## Abstract

**Importance:**

Patients prescribed long-term opioid therapy are increasingly undergoing dose tapering. Recent studies suggest that tapering is associated with short-term risks of substance misuse, overdose, and mental health crisis, although lower opioid dose could reduce risks of adverse events over the longer term.

**Objective:**

To assess the longer-term risks of overdose or mental health crisis associated with opioid dose tapering.

**Design, Setting, and Participants:**

This is a cohort study using an exposure-crossover analysis. Data were obtained from the OptumLabs Data Warehouse, which includes deidentified medical and pharmacy claims and enrollment records for commercial insurance and Medicare Advantage enrollees, representing a diverse mixture of ages, races, ethnicities, and geographical regions across the US. Participants were US adults who underwent opioid dose tapering from 2008 to 2017 after a 12-month baseline period of stable daily dosing of 50 morphine milligram equivalents or higher and who had at least 1 month of long-term follow-up during a postinduction period beginning 12 months after taper initiation. Data analysis was performed from October 2021 to April 2022.

**Exposures:**

Opioid tapering, defined as 15% or more relative reduction in mean daily dose during any of 6 overlapping 60-day windows within a 7-month follow-up period after the stable baseline period.

**Main Outcomes and Measures:**

Emergency or hospital encounters for drug overdose or withdrawal and mental health crisis (depression, anxiety, or suicide attempt). Outcome counts were assessed in pretaper and postinduction periods (from 12 to 24 months after taper initiation).

**Results:**

The study included 21 515 tapering events among 19 377 patients with a mean (SD) of 9.1 (2.7) months of postinduction follow-up per event (median [IQR], 10 [8-11] months). Patients had a mean (SD) age of 56.9 (11.2) years, 11 581 (53.8%) were female, and 8217 (38.2%) had commercial insurance (vs Medicare Advantage). In conditional negative binomial regression analyses, adjusted incidence rate ratios for the postinduction period compared with the pretaper period were 1.57 (95% CI, 1.42-1.74) for overdose or withdrawal and 1.52 (95% CI, 1.35-1.71) for mental health crisis.

**Conclusions and Relevance:**

These findings suggest that opioid tapering was associated with increased rates of overdose, withdrawal, and mental health crisis extending up to 2 years after taper initiation.

## Key Points

**Question:**

Is opioid dose tapering associated with reduced longer-term risks of overdose, withdrawal, or mental health crisis in patients prescribed long-term opioids?

**Findings:**

In this cohort study of 19 377 patients, in a posttaper period (beginning at least 12 months and extending up to 24 months after taper initiation) vs the pretaper period, the adjusted incidence rate ratios were 1.57 for overdose-withdrawal and 1.52 for a mental health crisis. Both were significant.

**Meaning:**

These findings suggest that opioid dose tapering was associated increased risks of overdose-withdrawal and mental health crisis that persisted up to 2 years after taper initiation.

## Teaser

This cohort study assesses the longer-term risks of overdose or mental health crisis associated with opioid dose tapering.

## Introduction

Over the past 2 decades, millions of Americans have been prescribed opioids to manage chronic pain. Although supported by limited evidence, medical educators encouraged the practice of initiating and escalating opioid dosages to address uncontrolled chronic pain, and drug companies aggressively marketed opioids, leading to increases in opioid prescriptions and the number of patients prescribed long-term opioid therapy (LTOT).^1^ As opioid overdose deaths subsequently increased, state and regional policies have encouraged opioid deprescribing and dose reduction, or tapering, among patients prescribed LTOT. The frequency of tapering among patients increased substantially after publication of a Centers for Disease Control and Prevention (CDC) opioid prescribing guideline in 2016.^2^

According to a US Department of Health and Human Services (HHS) guideline,^3^ dose tapering in patients prescribed LTOT should be considered when the risks of dose continuation outweigh the benefits in terms of pain relief and functional improvement. A dose-response association has been observed between long-term opioids and overdose risk,^4,5^ prompting the CDC to caution clinicians about escalating daily opioid doses greater than 50 morphine milligram equivalents (MME).^6^ On the other hand, opioid tapering may also confer patient risks, including precipitated withdrawal, worsening pain, use of illicit opioids, depression, anxiety, and suicide.^7,8,9,10^ Research documenting risks associated with tapering, however, has generally examined periods near the time of initial dose reduction or discontinuation. It is conceivable that opioid dose reduction in patients prescribed LTOT may reduce patient risks of adverse events with longer-term follow-up.

We recently reported that opioid dose tapering was associated with higher rates of overdose and mental health crisis during a 1-year follow-up period in a cohort of patients prescribed stable doses of LTOT.^11^ In the current study, we used an exposure-crossover design to examine longer-term risks of these adverse events among patients who initiated tapers in the cohort.^12,13^ In an exposure-crossover study, patients serve as their own controls, and event rates are compared before and after patients transition from the unexposed to the exposed state (from pretaper to posttaper). We hypothesized that the posttapering period would be associated with a lower risk of overdose and mental health crisis vs the pretapering period.

## Methods

### Study Data and Setting

This cohort study used deidentified administrative claims data from the OptumLabs Data Warehouse. The database contains deidentified retrospective administrative data, including medical and pharmacy claims (with associated diagnosis codes) and eligibility information for commercial insurance and Medicare Advantage enrollees. The database contains longitudinal health information on patients representing a mix of ages, races, ethnicities, and US geographical regions.^14^ The institutional review board of the University of California determined this study is not human participants research because it involved the analysis of preexisting, deidentified data; thus, informed consent was not necessary in accordance with 45 CFR §46. This study followed the Strengthening the Reporting of Observational Studies in Epidemiology (STROBE) reporting guideline.

### Design and Participants

The study had an exposure-crossover design in which outcomes are compared before and after an exposure within patients.^12,13^ Patients were members of a cohort of adults who received an opioid prescription from 2008 to 2017 and had average daily opioid doses of greater than or equal to 50 MME during each month of the previous 12-month baseline period with stable doses across the period (ie, each mean monthly dose was within 10% of the mean monthly dose across the period).^11^ Patients were excluded if they had cancer, had received hospice or palliative care or prolonged nursing home care, or had been prescribed buprenorphine during the baseline period. For this study, we included only patients identified as having initiated dose reductions, or tapers, during postbaseline follow-up and who had at least 1 month of longer-term follow-up after a 12-month induction period beginning on the first month of tapering. To identify tapers, we used an algorithm that identified tapers on the basis of a 15% or more dose reduction relative to the stable baseline dose during 6 overlapping 60-day periods after cohort entry on the first date following the end of the 12-month baseline period of stable dosing. The algorithm for identifying tapering and its predictive validity have been described elsewhere.^2,15^ The sampling strategy allowed patients to contribute more than 1 tapering period across the study period.

**Figure 1.**
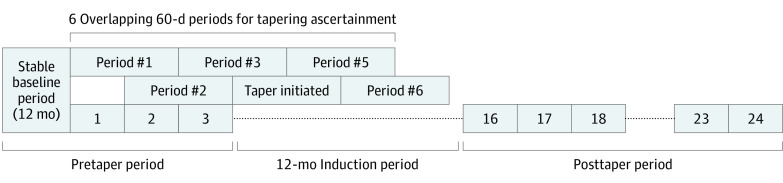
Illustration of Study Periods and Outcomes for Tapering Patient In this example of a patient who initiated tapering in month 4 after cohort entry, the baseline period and pretaper months (postbaseline months 1 to 3) are classified as the pretaper period. Subsequently, the patient’s 12-month induction period begins on month 4 and extends through month 15. The postinduction long-term follow-up period then begins on the 13th month after taper initiation and continues until the end of the study period (in this example, month 16 through month 24). For patients who initiate tapering earlier or later during follow-up, the pretaper and postinduction periods are adjusted accordingly.

In the exposure-crossover design, we compare outcome rates in pretaper and posttaper periods with patients serving as their own controls. Because patient characteristics are fixed across the 2 periods, the design has the advantage of controlling for fixed patient characteristics that may confound associations between tapering and adverse events. Nevertheless, prior studies^7,8,9,10,11^ suggest that the period of transition from the nontapered to tapered state is associated with elevated risk. We therefore included a 12-month induction period between the pretaper and posttaper periods to allow shorter-term risks associated with the transition to wash out and to identify longer-term risks associated with tapering. As illustrated in Figure 1, patient months included in the baseline period and pretaper months were classified as pretaper, and patient months occurring after the 12-month induction period were classified as postinduction. Patients were censored during the postinduction period because of health plan disenrollment, death, a new cancer diagnosis, or hospice or palliative care initiation.

### Outcomes

During pretaper and postinduction months (30-day periods), we used medical claims to specify counts of drug overdose and mental health crisis. We defined overdose or withdrawal events as emergency department visits or inpatient hospital admissions for any drug overdose, alcohol intoxication, or drug withdrawal. These events were identified by diagnosis codes included in the all-drug overdose definition specified in CDC drug overdose surveillance guidelines,^16^ in addition to codes for alcohol intoxication and alcohol or drug withdrawal. We also specified a narrower overdose events outcome using only the all-drug overdose codes (without additional alcohol or withdrawal codes). In validation studies^17,18^ based on medical record reviews, diagnosis codes for opioid overdoses identify emergency or hospital events for opioid overdose with positive predictive values ranging from 67% to 84%.

We defined mental health crisis events as emergency department or inpatient hospital admissions with depression or anxiety diagnosis codes in the primary diagnosis position or suicide attempt or intentional self-harm in any diagnosis position. In a systematic review,^19^ diagnostic codes for suicide attempts or intention had positive predictive values ranging from 55% to 100%. The list of diagnostic codes used to identify study outcomes is included in eTable 1 in the Supplement.

### Covariates

Sociodemographic information included age, sex, educational status (median education of adults aged ≥25 years in same US Census block), rurality of residence (dichotomized as metropolitan-micropolitan vs small town–rural using Rural-Urban Commuting Area codes),^20^ and insurance status (commercial vs Medicare Advantage). Baseline opioid dose, in MME per day, was calculated using pharmacy claims for all opioids during the baseline period (categorized as 50-89, 90-149, 150-299, and ≥300 MME). We assessed whether patients were prescribed a benzodiazepine (identified by National Drug Codes) on the date of cohort entry and a count of overdose events during the baseline year. Baseline depression or anxiety was identified by either claims diagnoses^21^ or having 1 or more pharmacy claim for a selective serotonin-reuptake inhibitor prescription during the baseline year. We assessed comorbidities using the Elixhauser Comorbidity Index, which includes variables labeled “alcohol abuse” and “drug abuse” (hereafter referred to as alcohol and drug use disorder, respectively).^22^

### Statistical Analysis

 Data analysis was performed from October 2021 to April 2022. Analyses were conducted using Stata MP statistical software version 15.1 (StataCorp). We performed descriptive analyses to characterize those patients identified as tapering from the original cohort and the fraction included in the exposure-crossover analysis (with at least 1 month of postinduction follow-up). In the exposure-crossover analyses, the time unit of analyses was the person-month. We first compared unadjusted incidence rates of study outcomes in the pretaper and postinduction periods with incidence rate differences, incidence rate ratios (IRRs), and 95% CIs. We then used conditional negative binomial regression to estimate adjusted IRRs of study outcomes in the postinduction period compared with the pretaper period, testing null hypotheses that within-participant rates of study outcomes did not differ in the 2 periods.^23^ Because patient-level covariates were fixed across the 2 periods, the conditional regression analysis statistically adjusts for fixed patient-level effects.

We performed 3 planned secondary analyses. First, we performed conditional negative binomial regression analyses with interaction terms between the pretaper vs postinduction indicator and baseline dose category (50-89, 90-149, 150-299, and ≥300 MME). We planned this analysis because patients prescribed higher baseline doses were at higher risk of adverse events during tapering during the initial year of follow-up.^11^ For each outcome, we performed Wald χ^2^ tests of the joint significance of the interaction terms and computed adjusted IRRs within baseline dose categories.

Second, we performed conditional negative binomial regression analyses with the postinduction period exposure variable categorized according to mean opioid dose achieved compared with baseline during the first 60-day period of the postinduction period, classified as discontinued (0 MME), 1% to 49%, 50% to 84%, 85% to 114% (near baseline), and 115% or higher (dose increased). We planned this analysis because risk reductions associated with tapering may be contingent on the achievement of sustained lowering of opioid dosage compared with baseline.

Third, we repeated the regression analyses with the postinduction period exposure variable dichotomized as 13 to 16 vs 17 to 24 months after taper initiation. This analysis addressed the question of whether a longer induction period might modify estimated risks.

Finally, to test the robustness of the exposure-crossover analyses, we incorporated long-term outcomes data for patient-periods in the original cohort in which tapering was not identified. We then conducted negative binomial regression for study outcomes during the postinduction period (13-24 months after cohort entry) by initial tapering status and achieved opioid dose at the beginning of the postinduction period. Details of these analyses are described in the eAppendix in the Supplement. Hypothesis tests were 2-sided with an α = .05.

## Results

**Figure 2.**
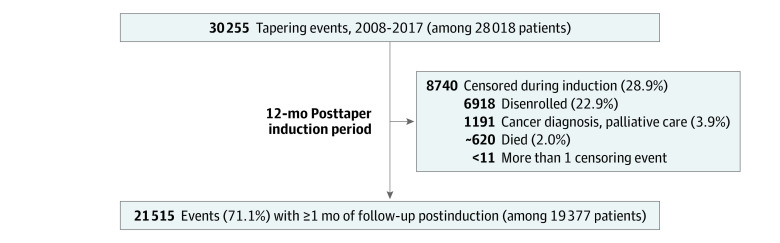
Flow Diagram of Tapering Events in Original Cohort and Postinduction Periods Some numbers are approximated to avoid small values of 10 or less.

From 2008 to 2017, there were 30 255 tapering events among 28 018 patients in the original cohort. Of these, 21 515 events (71.1%) among 19 377 patients were followed by at least 1 month of postinduction follow-up and were included in exposure-crossover analyses (Figure 2). At beginning of the postinduction follow-up, patients had a mean (SD) age of 56.9 (11.2) years, 11 581 (53.8%) were women, and 8217 (38.2%) had commercial insurance (vs Medicare Advantage). Among the 21 515 tapers with postinduction follow-up, the mean (SD) opioid dose relative to baseline during the first 60-day postinduction period was 0.61 (0.41), representing a mean relative dose reduction of 39%; by the first postinduction period, 3269 tapers (15.2%) resulted in opioid discontinuation, 5011 (23.3%) achieved a dose of 1% to 49% of baseline, 6869 (31.9%) achieved a dose of 50% to 84% of baseline, 5362 (24.9%) achieved a dose of 85% to 114% of baseline, and 1004 (4.7%) had a dose increase to greater than or equal to 115% of baseline. The mean (SD) duration of postinduction follow-up per taper event was 9.1 (2.7) months (median [IQR], 10 [8-11] months). The characteristics of patients included in the original cohort and the exposure-crossover analysis were similar (Table 1).

**Table 1.** Baseline Characteristics of Tapering Patients in Inception Cohort and Among Those With Postinduction Follow-up^a^

Characteristic	Patients, No. (%)	
	Tapering events in inception cohort (30 255 tapers among 28 018 patients)	Tapering events with postinduction follow-up (21 515 tapers among 19 377 patients)
Age category, y		
18-34.9	962 (3.2)	566 (2.6)
35-49.9	6437 (21.3)	4449 (20.7)
50-64.9	15 717 (52.0)	11 479 (53.4)
≥65	7139 (26.9)	5021 (23.3)
Sex		
Female	16 309 (53.9)	11 581 (53.8)
Male	13 946 (46.1)	9934 (46.2)
Education^b^		
High school or less	13 560 (44.8)	9737 (45.3)
More than high school	14 886 (49.2)	10 465 (48.6)
Unknown or missing	1809 (6.0)	1313 (6.1)
Rural vs urban residence^c^		
Metropolitan or micropolitan	28 245 (93.4)	20 044 (93.2)
Small town or rural	1933 (6.4)	1421 (6.6)
Missing	77 (0.3)	50 (0.2)
Commercial insurance	12 820 (42.4)	8217 (38.2)
Elixhauser comorbidities^d^		
Alcohol use disorder	738 (2.4)	483 (2.2)
Drug use disorder	4301 (14.2)	3030 (14.1)
Baseline opioid dose, MME/d		
50 to <90	6848 (22.6)	4879 (22.7)
90 to <150	7200 (23.8)	5126 (23.8)
150 to <300	9544 (31.6)	6767 (31.5)
≥300	6663 (22.0)	4743 (22.0)
Coprescribed benzodiazepine^e^	9078 (30.0)	6371 (29.6)
Baseline year overdose events^f^		
0	29 502 (97.5)	21 030 (97.8)
1	625 (2.1)	406 (1.9)
≥2	128 (0.4)	79 (0.4)
Baseline depression or anxiety^g^	16 310 (53.9)	11 613 (54.0)

Abbreviation: MME, morphine milliequivalents.

^a^ Patients with tapering events in original cohort and patients with long-term follow-up both had means of 1.1 baseline periods followed by tapering events (median [range], 1.0 [1.0-4.0]). Patient characteristics at end of included baseline periods are shown, and some patients are represented by more than 1 period.

^b^ Education was estimated according to median education level of resident aged 25 years or older in patient’s US Census block.

^c^ Rurality was derived from Rural-Urban Commuting Area codes.

^d^ Elixhauser comorbidities included 27 noncancer conditions, including alcohol use disorder and drug use disorder. The depression Elixhauser indicator was not included because of its redundancy with the preexisting depression or anxiety variable.

^e^ Coprescribed benzodiazepine was defined as a concurrent benzodiazepine prescription on the date of cohort entry.

^f^ Baseline overdose events were defined by specified diagnosis codes identified on emergency department or hospital claims in baseline year.

^g^ Baseline depression or anxiety was defined by specified diagnoses identified on emergency department, hospital, or outpatient claims, or pharmacy claims for selective serotonin-reuptake inhibitor during baseline year.

Although we hypothesized that tapered patients would have reduced rates of study outcomes during the postinduction period compared with the pretaper period, unadjusted and adjusted incidence rates of overdose or withdrawal, overdose, and mental health crisis were each increased significantly in the postinduction period compared with the pretaper period (Table 2). In conditional negative binomial regression analyses, adjusted IRRs for the postinduction period compared with the pretaper period were 1.57 (95% CI, 1.42-1.74) for overdose or withdrawal, 1.40 (95% CI, 1.22-1.61) for overdose, and 1.52 (95% CI, 1.35-1.71) for mental health crisis (Table 2).

**Table 2.** Incidence and IRRs of Study Outcomes by Pretaper and Postinduction Periods Among Patients Who Underwent Opioid Dose Tapering^a^

Outcome	Pretaper		Postinduction		IRD/100 person-years (95% CI)^b^	IRR (95% CI)^b^	
	Events, No./person-years, No.	Incidence rate, events/100 person-years	Events, No./person-years, No.	Incidence rate, events/ 100 person-years		Unadjusted	Adjusted^c^
Overdose or withdrawal	888/25 142	3.5	880/16 257	5.4	1.9 (1.5-2.3)	1.53 (1.39-1.68)	1.57 (1.42-1.74)
Overdose	505/25 142	2.0	455/16 257	2.8	0.8 (0.5 1.1)	1.39 (1.22-1.58)	1.40 (1.22-1.61)
Mental health crisis	747/25 142	3.0	714/16 257	4.4	1.4 (1.0-1.8)	1.48 (1.33-1.64)	1.52 (1.35-1.71)

Abbreviations: IRD, incidence rate difference; IRR, incidence rate ratio.

^a^ There were 21 515 tapers among 19 377 patients.

^b^ IRDs and IRRs are all significant with *P* < .001.

^c^ Estimated using fixed-effects negative binomial regression.

Table 3 shows adjusted IRRs associated with the postinduction period compared with the pretaper period among patients by baseline dose. For all 3 outcomes, adjusted IRRs for postinduction adverse events were highest for patients in the highest baseline dose group (≥300 MME). Tests for heterogeneity in tapering IRRs across baseline dose groups were significant for overdose-withdrawal and mental health crisis but not for overdose alone.

**Table 3.** Adjusted IRRs of Overdose or Mental Health Crisis in the Postinduction Compared With the Pretaper Period by Patient or Period Subgroups^a^

Patient or period subgroup	Overdose or withdrawal		Overdose		Mental health crisis	
	IRR (95% CI)	*P* value^b^	IRR (95% CI)	*P* value^b^	IRR (95% CI)	*P* value^b^
Baseline dose, MME^c^						
50-89	1.24 (0.98-1.58)	.01	1.04 (0.75-1.44)	.15	1.26 (0.97-1.63)	<.001
90-149	1.54 (1.24-1.90)		1.43 (1.08-1.91)		1.18 (0.93-1.49)	
150-299	1.47 (1.23-1.75)		1.40 (1.11-1.76)		1.49 (1.21-1.82)	
≥300	2.03 (1.67-2.47)		1.71 (1.31-2.24)		2.54 (1.95-3.30)	
Postinduction achieved dose vs baseline^d^						
Discontinued	1.09 (0.88-1.36)	<.001	0.86 (0.62-1.20)	<.001	1.17 (0.91-1.50)	.13
1%-49%	1.32 (1.08-1.61)		1.07 (0.82-1.39)		1.58 (1.26-1.97)	
50%-84%	1.93 (1.61-2.32)		1.86 (1.46-2.37)		1.77 (1.43-2.19)	
85%-114%	2.16 (1.71-2.73)		1.93 (1.43-2.62)		1.59 (1.23-2.06)	
≥115%	1.56 (1.00-2.43)		1.64 (0.94-2.87)		1.28 (0.76-2.16)	
Early vs later in postinduction period						
Early (months 13-16)	1.56 (1.32-1.84)	.94	1.32 (1.05-1.67)	.53	1.56 (1.28-1.89)	.77
Later (months 17-24)	1.57 (1.41-1.75)		1.42 (1.24-1.64)		1.51 (1.33-1.71)	

Abbreviations: IRR, incidence rate ratio; MME, morphine milliequivalents.

^a^ Data were estimated using fixed-effects negative binomial regression (21 515 tapers among 19 377 patients).

^b^ *P* values are for χ^2^ tests for significant heterogeneity in IRRs across subgroups.

^c^ Stratum-specific IRRs were estimated by fitting models with interaction terms between pretaper vs posttaper period and baseline dose categories.

^d^ Defined as the average opioid dose (in MME) during the first postinduction 60-day period divided by the average opioid dose during the 12-month stable baseline period.

In analyses stratified by postinduction achieved dose (Table 3), adjusted IRRs of overdose-withdrawal and overdose during the postinduction period compared with the pretaper period were greater among patients whose postinduction doses were 50% to 84% or 85% to 114% of their baseline doses compared with patients who discontinued opioids or sustained a dose reduction of 1% to 49% of baseline. For example, the adjusted IRR of overdose was 0.86 (95% CI, 0.62-1.20) among tapers with opioid discontinuation compared with 1.93 (95% CI, 1.43-2.62) among tapers with an achieved dose of 85% to 114% of baseline. For both overdose-withdrawal and overdose, there was significant heterogeneity in adjusted IRRs for tapering by achieved postinduction dose. For mental health crises, there was no significant difference in adjusted IRRs by achieved dose.

Adjusted IRRs of all 3 outcomes were similar earlier compared with later in the postinduction period (Table 3). Results of planned secondary analyses were largely similar in unadjusted analyses (eTable 2 in the Supplement). In sensitivity analyses that incorporated long-term outcome data for patient-periods from the original cohort without initial tapering, initial tapering after cohort entry remained significantly associated with overdose-withdrawal, overdose, and mental health crisis during the postinduction period (eTable 3 in the Supplement).

## Discussion

During the initial 1-year follow-up period of this study’s original cohort,^11^ we observed increased rates of overdose and mental health crisis after opioid dose tapering. In the current cohort study using an exposure-crossover analysis, we extend these findings by examining rates for these adverse events among the tapered patients starting a year after tapering and up to 24 months after the baseline year of stable dosing. Our results suggest that the increased rates of overdose and mental health crisis observed during the first year after tapering persist through the end of the 24-month follow-up.

We had hypothesized that opioid dose reduction would be associated with reduced longer-term rates of overdose and mental health crises. Although patients may struggle during the tapering period, we reasoned that many may stabilize with longer-term follow-up and have lower rates of overdose and mental health crisis once a lower opioid dose is achieved. Although evidence has accumulated that opioid tapering may pose risks,^8,9,24^ particularly if doses are precipitously reduced or discontinued,^11^ small studies^25,26,27^ suggest that sufficiently supported patients can safely achieve opioid dose reduction with potential improvements in patient pain, function, and quality of life. In the current study, no achieved tapered dose was associated with significantly reduced posttaper rates of adverse events compared with the pretaper period, although relative rates of adverse events were similar in the baseline and postinduction periods among the 15.2% of tapers that resulted in opioid discontinuation. Nevertheless, our findings suggest that, for most tapering patients, elevated risks of overdose and mental health crisis may persist for up to 2 years after taper initiation.

A contributing factor may be the study period, which includes tapering events initiated from 2008 to 2017. Clinicians supervising tapers during this period may have been unaware of recommended practices to increase the safety of tapering, which may not have been widely disseminated to clinicians until 2019 with the publication of the HHS guideline on appropriate opioid dose reduction.^3^ We also recognize the possibility of within-participant confounding, as patients with stable baseline dosing who then undergo tapering may be selected for tapering because of unmeasured risk factors that emerge around the time of tapering and then heighten longer-term patient risk of overdose or mental health crisis. Nevertheless, it is possible that dose variability in opioid-dependent patients poses durable risks of substance misuse or mental health deterioration,^9,28^ which could heighten longer-term patient risk of overdose and mental health crisis after dose tapering.

In earlier work,^11^ patients prescribed higher baseline opioid doses had higher absolute rates of overdose and mental health crises associated with tapering. The current study suggests that higher baseline dose is also associated with greater longer-term risk of overdose-withdrawal and mental health crisis. HHS tapering guidelines^3^ emphasize close follow-up, monitoring for substance use and mental health deterioration, and psychosocial support for patients undergoing opioid tapering after long-term use. Although our results suggest that all tapering patients may benefit from monitoring and support up to 2 years after taper initiation, patients prescribed higher doses may benefit from more intensive support and monitoring, particularly for depression and suicidality.

### Limitations

The limitations of this study warrant consideration. First, although the within-participant design controls for fixed patient covariates, it may be vulnerable to confounding by patient-level factors that change over time, including potential risk factors for overdose or mental health crisis that emerge within patients during the 3-year observation window of each tapering event. Second, exposure-crossover studies are potentially vulnerable to confounding secular trends, although the postinduction and baseline periods of each tapering event were separated by a short, 12-month interval. In addition, initial tapering remained associated with higher rates of overdose and mental health crisis during the postinduction period in sensitivity analyses that adjusted for study year and incorporated data from patients who did not initially taper. Third, it is conceivable that the 12-month induction period was too short to allow the initial impacts of tapering to wash out, although we found similar adjusted IRRs when categorizing the follow-up period as earlier vs later during the induction period. Fourth, the sample derived from a data source representing patients with commercial insurance or Medicare Advantage, and results may not be generalizable to other populations. Fifth, a considerable fraction of patients were censored before the postinduction follow-up, which could have introduced systematic bias. Sixth, opioid tapering was identified on the basis of dose reductions identified in pharmacy claims. Although most patients with initial tapers achieved lower doses by the beginning of the induction period, the claims data do not yield insight into the reasons for the dose reduction or whether it was patient or clinician initiated. Seventh, study events were identified using diagnostic codes on claims, which may lack sensitivity or specificity, resulting in measurement error.

## Conclusions

In this cohort study using an exposure-crossover analysis that controls for between-person effects, opioid dose tapering was associated with persistently elevated risk of overdose, withdrawal, and mental health crisis up to 24 months after taper initiation. Given the observational study design, we cannot infer a causal connection between tapering and long-term risks of these events. Nevertheless, our findings support guidelines advising careful monitoring and psychosocial support for patients undergoing opioid dose reduction and suggest that this support continue for at least 2 years after taper initiation, particularly among patients who were prescribed higher baseline doses.

## Notes

Supplement. **eAppendix. **Supplemental Methods **eTable 1. **Outcome Definitions by *International Classification of Diseases, Clinical Modification* Codes **eTable 2.** Unadjusted Incidence Rate Ratios of Outcomes in Postinduction Relative to Pretaper Periods by Patient Subgroup or Subperiod **eTable 3.** Negative Binomial Regression Analyses of Overdose and Mental Health Crisis Outcomes Occurring 13 to 24 Months After Cohort Entry Among Patients by Initial Tapering Status and Achieved Opioid Dose Postinduction Period (71,687 Patients and 96,358 Person-years)
eReference
