# Error in Third Author’s Name

**DOI:** 10.1001/jamanetworkopen.2022.24285

**Published:** 2022-07-15

In the Original Investigation titled “Long-term Risk of Overdose or Mental Health Crisis After Opioid Dose Tapering,”^1^ published June 13, 2022, the third author’s first name was misspelled. This article has been corrected.^1^
