# Supplementary material for: Long-term Risk of Overdose or Mental Health Crisis After Opioid Dose Tapering
Source: JAMA Netw Open. 2022 Jun 13;5(6):e2216726. doi: 10.1001/jamanetworkopen.2022.16726 (PMC9194670; doi:10.1001/jamanetworkopen.2022.16726)
Supplement: Supplement. — eAppendix. Supplemental Methods eTable 1. Outcome Definitions by International Classification of Diseases, Clinical Modification Codes eTable 2. Unadjusted Incidence Rate Ratios of Outcomes in Postinduction Relative to Pretaper Periods by Patient Subgroup or Subperiod eTable 3. Negative Binomial Regression Analyses of Overdose and Mental Health Crisis Outcomes Occurring 13 to 24 Months After Cohort Entry Among Patients by Initial Tapering Status and Achieved Opioid Dose Postinduction Period (71,687 Patients and 96,358 Person-years) eReference [file jamanetwopen-e2216726-s001.pdf]

Supplemental Online Content

Fenton JJ, Magnan E, Tseregounis IE, Xing G, Agnoli AL, Tancredi DJ. Long-term risk of overdose or mental health crisis after opioid dose tapering. *JAMA Netw Open*. 2022;5(6):e2216726. doi:10.1001/jamanetworkopen.2022.16726

eAppendix. Supplemental Methods

eTable 1. Outcome Definitions by *International Classification of Diseases, Clinical Modification* Codes

eTable 2. Unadjusted Incidence Rate Ratios of Outcomes in Postinduction Relative to Pretaper Periods by Patient Subgroup or Subperiod

eTable 3. Negative Binomial Regression Analyses of Overdose and Mental Health Crisis Outcomes Occurring 13 to 24 Months After Cohort Entry Among Patients by Initial Tapering Status and Achieved Opioid Dose Postinduction Period (71,687 Patients and 96,358 Person-years)

eReference

This supplemental material has been provided by the authors to give readers additional information about their work.

© 2022 Fenton JJ et al. *JAMA Network Open.*

eAppendix. Supplemental Methods

Sensitivity Analyses

To assess the robustness of the within-subject, self-controlled design, we performed sensitivity analyses that incorporated data on longer-term outcomes after patient-periods without initial tapering identified in the original cohort analyses.[1] In analyses of outcomes up to one year of follow-up, patient-periods without tapering served as a "control" comparison for patient-periods after tapering was identified.

In the sensitivity analyses, we sought to compare longer-term outcomes (in months 13-24) among patients identified as initially tapered and not tapered. Among tapered patients, longer-term follow-up commenced after a 12-month post-taper induction period. So that the distribution of follow-up times would be similar after tapered and non-tapered patient-periods, all non-tapered patient periods were randomly assigned a starting month of the 12-month induction period based on the distribution of the initial tapering months in the tapered patient-periods. We then ascertained monthly counts of overdose and mental health crisis outcomes after the 12-month induction period in the non-tapered patients. Of 166,750 patient periods without initial tapering (among 96,143 patients) that were included in the original cohort,[1] 105,692 patient periods without initial tapering (among 52,310 patients) had at least one month of post-induction follow-up and were included in these sensitivity analyses. Analyses also included all initially tapered patients that were analyzed in the exposure-crossover analyses (21,515 periods in 19,377 patients).

For each outcome, we conducted two sensitivity analyses. First, we used negative binomial regression to model counts of study outcomes during post-induction months by initial tapering status after cohort entry (Model #1). Second, we conducted a similar negative binomial analysis by both initial tapering status and the achieved post-induction opioid dose among patients who did not initially taper (Model #2). Tapering status in this second analysis was categorized as: 1) initially tapered; 2) not initially tapered, discontinued opioids post-induction; 3) not initially tapered, post-induction dose 1-49% of baseline; 4) not initially tapered, post-induction dose 50-84% of baseline; 5) not initially tapered, post-induction dose 85-114% of baseline; 6) not initially tapered, post-induction dose increased (≥115% of baseline). Because the fifth category comprised patients with stable dosing both initially and at the beginning of the post-induction period, this group was chosen as the referent for the analyses. Among patients without initial tapering, the achieved post-induction dose was distributed as: discontinued (4.1%), 1-49% of baseline (4.7%), 50-84% of baseline (10.5%), 85-114% of baseline (66.2%), and increased to ≥115% of baseline (14.4%).

All models adjusted for age, sex, education, rural vs. urban residence, commercial vs. Medicare Advantage insurance, co-prescription of benzodiazepines on cohort entry, a count of overdose events during the baseline year, a count of mental health crisis events during the baseline year, baseline depression or anxiety (defined by diagnoses for depression, anxiety or suicide on claims, or pharmacy claims for selective serotonin reuptake inhibitors during the baseline year), indicators for 27 non-cancer comorbidities identified in the Elixhauser index, and study year. We used robust standard errors to account for clustering of study months within patients. As shown in eTable 3, both initial tapering and subsequent dose reduction were associated with increased incidence rate ratios for study outcomes during the post-induction period occurring 13-24 months after cohort entry.

eTable 1. Outcome Definitions by *International Classification of Diseases, Clinical Modification* Codes

Outcome	Components	ICD-9-CM codes	ICD-10-CM codes
Overdose/Withdrawal	All-drug overdose	960-979; E850-858; E950.0-E950.5; E962.0; E980.0-E980.5.	T36-T50 (with 5th or 6th character of 1-4, and 7th character of A or missing – specifying intentionality or accidental and initial encounter).
	Opioid withdrawal or alcohol intoxication or withdrawal	303.0, 303.9, 305.0, 292.0 (with 304.0 or 303.9).	F10.10, F10.11, F10.12, F10.14, F10.15, F10.2, F10.21, F10.22, F10.24, F10.25x, F10.26, F10.27, F10.28x, F10.29, F10.9x; F11.23, F11.93, F10.23x
Overdose	All-drug overdose	960-979; E850-858; E950.0-E950.5; E962.0; E980.0-E980.5.	T36-T50 (with 5th or 6th character of 1-4, and 7th character of A or missing – specifying intentionality or accidental and initial encounter).
Mental Health Crisis	Depression	296.20-296.26, 296.30-296.36, 296.51-296.56, 296.60-296.66, 296.89, 298.0, 300.4, 309.1, 311	F31.30-F31.32, F31.4, F31.5, F31.60-F31.64, F31.75-F31.78, F31.81, F32.0-F32.5, F32.9, F33.xx, F34.1, F43.21, F43.23
	Anxiety	293.84, 300.0x, 300.10, 300.20-300.22, 300.3, 300.5, 300.89, 300.9, 308.x, 309.81	F06.4, F40.0x, F41.0, F41.x, F42.x, F43.x, F44.9, F45.8, F48.8, F48.9, F99, R45.2, R45.5, R45.6, R45.7
	Suicide attempt	E950-959	T14.91, T40.0X2, T40.1X2, T40.2X2, T40.3X2, T40.4X2, T40.5X2, T40.602, T40.692, X71-83

Abbreviations: ICD-9-CM=International Classification of Diseases, 9th Revision, Clinical Modificatio,; ICD-10-CM= International Classification of Diseases, 10th Revision, Clinical Modification

eTable 2. Unadjusted Incidence Rate Ratios of Outcomes in Postinduction Relative to Pretaper Periods by Patient Subgroup or Subperiod

Patient or period subgroup	Overdose or withdrawal	Overdose	Mental health crisis
	Unadjusted IRR (95% CI)	Unadjusted IRR (95% CI)	Unadjusted IRR (95% CI)
**Baseline Dose (in MME)**			
50-89	1.25 (0.99, 1.58)	1.13 (0.82, 1.55)	1.28 (1.01, 1.62)
90-149	1.59 (1.30, 1.95)	1.50 (1.14, 1.98)	1.17 (0.95, 1.44)
150-299	1.34 (1.14, 1.58)	1.33 (1.06, 1.65)	1.39 (1.16, 1.67)
≥300	1.97 (1.64, 2.38)	1.59 (1.23, 2.07)	2.48 (1.96, 3.15)
**Post-induction achieved dose (relative to baseline)[b]**			
Discontinued	1.02 (0.82, 1.25)	0.81 (0.59, 1.12)	1.10 (0.88, 1.38)
1-49%	1.30 (1.08, 1.57)	1.09 (0.84-1.41)	1.56 (1.27, 1.91)
50-84%	1.91 (1.60, 2.28)	1.80 (1.42, 2.30)	1.83 (1.50, 2.23)
85-114%	2.19 (1.74, 2.76)	1.91 (1.42, 2.58)	1.52 (1.19, 1.95)
>=115%	1.92 (1.23, 3.02)	2.21 (1.25, 3.99)	1.20 (0.72, 1.99)
**With induction period categorized as early vs. later**			
Early post-induction period (months 13-16)	1.95 (1.79, 2.11)	1.92 (1.73, 2.14)	1.88 (1.71, 2.08)
Later post-induction period (months 17-24)	1.95 (1.84, 2.07)	1.81 (1.68, 1.96)	1.79 (1.66, 1.92)

© 2022 Fenton JJ et al. *JAMA Network Open.*

eTable 3. Negative Binomial Regression Analyses of Overdose and Mental Health Crisis Outcomes Occurring 13 to 24 Months After Cohort Entry Among Patients by Initial Tapering Status and Achieved Opioid Dose Postinduction Period (71,687 Patients and 96,358 Person-years)

Specification of tapering status	Overdose/Withdrawal	Overdose	Mental Health Crisis
	IRR (95% CI) [a]	IRR (95% CI) [a]	IRR (95% CI) [a]
**Model #1: Based on initial tapering status[b]**			
Not initially tapered	1.0 (ref)	1.0 (ref)	1.0 (ref)
Initially tapered	1.18 (1.07, 1.29)	1.14 (1.01-1.30)	1.19 (1.06-1.33)
**Model #2: Based on initial tapering status and achieved post-induction opioid dose if not initially tapered[c]**			
Initially tapered	1.38 (1.25, 1.53)	1.34 (1.17, 1.53)	1.51 (1.33, 1.71)
No initial taper, discontinued post-induction	1.27 (1.04, 1.56)	1.26 (0.97, 1.64)	2.03 (1.58, 2.62)
No initial taper, 1-49% of baseline post-induction	1.79 (1.50, 2.13)	1.62 (1.30, 2.02)	2.05 (1.66, 2.54)
No initial taper, 50-84% of baseline post-induction	1.46 (1.29, 1.66)	1.42 (1.21, 1.66)	1.91 (1.63, 2.23)
No initial taper, 85-114% of baseline post-induction[d]	1.0 (ref)	1.0 (ref)	1.0 (ref)
No initial taper, dose increased post-induction (>=115% of baseline)	1.46 (1.29, 1.65)	1.56 (1.34, 1.83)	1.44 (1.22, 1.69)

[a] Incidence rate ratios (IRRs) adjusted for age, sex, education, rural vs. urban residence, commercial vs. Medicare Advantage insurance, co-prescription of benzodiazepines on cohort entry, a count of overdose events during the baseline year, a count of mental health crisis events during the baseline year, baseline depression or anxiety (defined by diagnoses for depression, anxiety or suicide on claims, or pharmacy claims for selective serotonin reuptake inhibitors during the baseline year), indicators for 27 non-cancer comorbidities identified in the Elixhauser index, and study year.

[b] Initial tapering status ascertained based on ≥15% dose reduction relative to baseline in the initial six overlapping 60-day periods after cohort entry. Outcomes ascertained during follow-up months beginning 12 months after tapering identified. For patient-periods without tapering, the beginning of the period of outcome ascertainment was randomly assigned based on the distribution of initial follow-up months in patient-periods with tapering.

[c] Initial tapering ascertained as above based on relative dose reduction in the initial six overlapping 60-day periods after cohort entry. If no initial taper, patients were classified based on the average opioid dose relative to baseline during the first 60-day period after a 12-month induction period. The induction period among patients without initial tapers began on a randomly assigned starting month based on the distribution of post-induction starting months in initially tapered patients.

[d] Patients without initial tapering and whose achieved dose was 85-114% of baseline were chosen as the referent group because these patients had relative dose stability during the initial six overlapping 60-day periods after cohort entry and at the beginning of the post-induction period which occurred at months 13-18 of follow-up.

© 2022 Fenton JJ et al. *JAMA Network Open.*

eReference

1. Agnoli A, Xing G, Tancredi DJ, Magnan E, Jerant A, Fenton JJ. Association of Dose Tapering With Overdose or Mental Health Crisis Among Patients Prescribed Long-term Opioids. *JAMA*. 2021;326(5):411-419. doi:10.1001/jama.2021.11013

© 2022 Fenton JJ et al. *JAMA Network Open.*
